# Comprehensive Characterization of Ageing-Relevant Subtypes Associated With Different Tumorigenesis and Tumor Microenvironment in Prostate Cancer

**DOI:** 10.3389/fmolb.2022.803474

**Published:** 2022-02-21

**Authors:** Liang Huang, Zhenzhou Xu, Yu Xie, Shusuan Jiang, Weiqing Han, Zhengyan Tang, Quan Zhu

**Affiliations:** ^1^ Department of Urology, the Affiliated Cancer Hospital of Xiangya School of Medicine, Central South University, Hunan Cancer Hospital, Changsha, China; ^2^ Department of Urology, Xiangya Hospital, Central South University, Changsha, China

**Keywords:** prostate cancer, ageing, subtype, gene signature, tumor microenvironment, prognosis

## Abstract

**Objective:** Accumulated evidence demonstrates that ageing is a robust risk factor of prostate cancer prognosis. Herein, we conducted a systematic analysis about ageing-relevant molecules and relevant tumor microenvironment features in prostate cancer.

**Methods:** Transcriptome data, clinical information, and mutational data of prostate cancer patients were retrospectively collected from the Cancer Genome Atlas cohort. In accordance with the expression of specific ageing-relevant genes, prostate cancer patients were clustered with consensus clustering analyses. WGCNA was adopted for determination of subtype-associated co-expression modules and genes. Thereafter, characteristic genes were further screened with random forest algorithm and a prognostic model was conducted with multivariate cox regression analyses. Tumor microenvironment-infiltrating immune cells were estimated with ssGSEA and ESTIMATE. Activities of the cancer immunity cycle and expressions of HLA and immune checkpoint molecules were then quantified across prostate cancer cases. A serious experiment was conducted to investigate the roles of EIF2S2 in prostate tumorigenesis.

**Results:** This study characterized three ageing-relevant subtypes (C1, C2, and C3) with diverse clinical prognosis. Subtype C1 presented the features of low mutational frequency and immune activation; C2 was characterized by stromal and immune activation; and C3 showed immune suppression. An ageing-derived gene signature was conducted, which independently and robustly predicted patients’ prognosis. Additionally, this signature was in relation to immune inactivation. Among the genes in the signature, EIF2S2 triggered proliferation, invasion, and migration of LNCaP and PC-3 cells.

**Conclusion:** Collectively, ageing-relevant molecular subtypes and gene signature might be of great significance to determine clinical outcomes and tumor microenvironment features and immunotherapeutic responses in prostate cancer.

## Introduction

Prostate cancer is a frequent malignant tumor of the male genitourinary system across the globe (Culp et al., 2020). As estimated, it influences 1/9 men in America and is the second major cause of cancer-related deaths (Swami et al., 2020). It represents a heterogeneous malignancy in accordance with clinical, morphological, and molecular levels (Haffner et al., 2021). Clinical manifestations range from locally indolent to rapidly progressing fatal metastatic malignancy. Though most men are diagnosed with organ-limited malignancy, long-period oncology results may hugely vary (Wang et al., 2018). Moreover, histomorphology and molecular tumor features show prominent diversity between diverse patients and within a specific tumor. About 20% of patients with localized prostate cancer will develop regional or distant metastases (Buckup et al., 2021). Antiandrogen therapy has become the standardized treatment regimen for advanced or metastatic patients (Ge et al., 2020). Nevertheless, most patients will develop castration resistance. Thus, it is of significance to develop an effective risk stratification as well as favorable therapeutic agents against prostate cancer.

Ageing is a process which features a gradual loss of physiological integrity, contributing to impaired functions as well as increased vulnerability to death (Calcinotto et al., 2019). This process is regarded as a dominating risk factor of prostate cancer (VanderWalde and Hurria, 2011). Growing evidence demonstrates that ageing is related to telomere shortening, mitochondrial dysfunctions, DNA injury, immune system disorders and the like, and may be suppressed through calorie restriction (Jia et al., 2018). Transcriptomic research has uncovered that ageing presents a prominent discrepancy at molecular levels (Jia et al., 2018). Currently, numerous human ageing-relevant genes have been identified in several cancer types (de Magalhães et al., 2005). For instance, a prognostic aging-relevant gene signature has been conducted in head and neck squamous cell carcinoma, which is related to immunosuppressive state as well as inflammatory response (Yang et al., 2020). Nevertheless, features of these ageing-relevant genes in prostate cancer remain indistinct. To comprehensively analyze these specific genes might assist in deepening the comprehension of the ageing process as well as offering worthy clues concerning intervention strategies in prostate cancer. Herein, this study systematically dissected the features of transcriptional ageing-relevant genes from diverse perspectives.

## Materials and Methods

### Prostate Cancer Data Sets and Preprocessing

Transcriptome data (fragments per kilobase million (FPKM) value), clinical information and mutational data of prostate cancer were retrieved from the Cancer Genome Atlas (TCGA) data portal (https://portal.gdc.cancer.gov/). Thereafter, the FPKM were transformed to transcripts per million (TPM). Microarray expression profiling and matched survival information of 248 prostate cancer patients was retrieved from the Gene Expression Omnibus (GEO) repository (https://www.ncbi.nlm.nih.gov/gds/; accession number: GSE116918) (Jain et al., 2018). The raw data were preprocessed and normalized utilizing robust multichip average algorithm with affy package (version 1.72.0). The probes were transformed to gene symbols following the corresponding platform annotation file. Aging-relevant genes were retrieved from the human aging genome resource (HAGR; https://genomics.senescence.info/), which is a collection of online resources for studying the biology of human ageing on the basis of genetic perturbations in animal models and human diseases and extensive literature reviews (de Magalhães et al., 2005). The list of aging-relevant genes was listed in Supplementary Table S1.

### Differential Expression Analyses

Differentially expressed ageing-relevant genes were determined between prostate tumor and control specimens utilizing empirical Bayesian method of limma package (version 3.50.0) (Ritchie et al., 2015). Genes with an adjusted *p* value of < 0.05 were determined as prostate cancer-specific ageing-relevant genes.

### Unsupervised Clustering for Ageing-Relevant Genes

Univariate cox regression models were conducted for screening prognostic prostate cancer-specific ageing-relevant genes with *p* < 0.05. Following the expression of these genes, prostate cancer cases were classified into distinct subtypes with ConsensusClusterPlus package (version 1.58.0) (Wilkerson and Hayes, 2010) utilizing unsupervised clustering analyses. Principal component analyses (PCA) were conducted for visualization of dissimilarities among diverse clusters.

### Somatic Mutation and Copy-Number Alteration (SCNA) Analyses

Genetic mutation data in Mutation Annotation Format (MAF) of prostate cancer were retrieved from TCGA project. Through maftools package (version 2.10.0) (Mayakonda et al., 2018), somatic mutations were analyzed. The overall mutational status was conducted in each subtype. Moreover, GISTIC2.0 (Mermel et al., 2011) was applied for analyzing amplification and deletion across SCNA data that were curated from GDAC Firehose (https://gdac.broadinstitute.org).

### Quantification of Activities of Known Biological Processes

The gene sets of a few known biological processes were curated from Mariathasan et al. (Mariathasan et al., 2018). These biological processes contained epithelial-mesenchymal transition (EMT; EMT1, EMT2, and EMT3; immune checkpoint; antigen processing machinery (APM); CD8 + T effector; angiogenesis; and pan-fibroblast TGFβ response (pan-F-TBRS); DNA damage repair; FGFR3-related genes; KEGG discovered histones; Fanconi anemia; cell cycle; cell cycle regulators; DNA replication; nucleotide excision repair; homologous recombination; mismatch repair; and WNT target.

### Estimation of Tumor-Infiltrating Immune Cells

A single-sample gene-set enrichment analysis (ssGSEA) algorithm was adopted to quantify the relative abundance of tumor-infiltrating immune cells based on the expression profiling of 782 meta-genes (Charoentong et al., 2017) utilizing GSVA package (version 1.42.0) (Hänzelmann et al., 2013) across cases of prostate cancer. Through the scale algorithm, the enrichment levels of tumor-infiltrating immune cells were normalized for in-depth analyses. Additionally, Estimation of STromal and Immune cells in MAlignant Tumours using Expression data (ESTIMATE) package was employed for quantification of the relative abundance of immune and stromal cell populations in prostate cancer transcriptome data (Yoshihara et al., 2013). Thereafter, tumor purity was inferred in each specimen.

### Weighted Gene Co-Expression Network Analyses (WGCNA)

The WGCNA method (version 1.4.0) (Langfelder and Horvath, 2008) can transform expression data into co-expression gene modules, as well as explore the interactions of co-expression modules with clinical phenotypes. Herein, WGCNA was conducted based on the expression profiles of prostate cancer. Scale independence and mean connectivity were calculated across different soft threshold (power) values (ranging from 1 to 20). The optimal soft threshold was determined in accordance with scale independence >0.85. Thereafter, genes were classified into diverse gene co-expression modules following topological overlap matrix (TOM)-based dissimilarity. The co-expression module with the highest association with phenotypes was regarded as the key module and genes in this module were screened as ageing-derived genes.

### Functional Enrichment Analyses

Through clusterProfiler package (version 4.2.1) (Yu et al., 2012), functional annotation analyses of ageing-derived genes were conducted, containing Gene Ontology (GO) as well as Kyoto Encyclopedia of Genes and Genomes (KEGG). Gene Set Enrichment Analyses (GSEA; version 4.0.2) (Subramanian et al., 2005) were adopted for ascertaining the diverse pathways between groups. The gene sets of “c2. cp.kegg.v6.2.-symbols” were curated from the Molecular Signatures database (MSigDB) project (Liberzon et al., 2015) for running GSVA enrichment analyses. *p* values < 0.05 indicated prominent differences in biological functions and pathways.

### Construction of an Ageing-Derived Gene Signature

Univariate cox regression models were applied for determining prognostic ageing-derived genes with *p* < 0.05. With the random forest algorithm, the most important genes were determined in accordance with the relative importance >0.2. An ageing-relevant gene signature was conducted through combining the expression of characteristic genes and regression coefficients derived from multivariate cox regression analyses.

### Cell Culture and Transfections

Human prostate cancer cell lines (LNCaP and PC-3; Chinese Academy of Sciences) were maintained in RPMI 1640 medium plus 10% fetal bovine serum (FBS; GIBCO, United States ). All cell lines were grown at 37°C in a humidified environment of 5% CO_2_. Small interference RNA against EIF2S2 (si-EIF2S2) and negative control (si-NC) were retrieved from GenePharma (Shanghai, China). Full-length EIF2S2 cDNAs were amplified and cloned into pcDNA3.1 express vectors. Non-targeting pcDNA3.1 express vectors were utilized as a control (empty vector). Transfections were presented via lipofectamine 2000 reagent in accordance with the manufacturer’s instructions.

### Western Blotting

Protein extracts were harvested from cell specimens in RIPA lysis buffer (Millipore, Germany) plus phosphatase inhibitor Cocktail III. The concentrations of protein specimens were quantified utilizing BCA kits (Thermo Fisher Scientific, United States ). In total, 20 μg protein was loaded onto a 10% SDS-PAGE gel as well as transferred to 0.22 μm PVDF membrane (Millipore, Germany). The membrane was sealed by 1 × TBST buffer plus 5% nonfat milk. Thereafter, the membrane was probed by specific antibodies targeting EIF2S2 (1:500; #10227-1-AP; Proteintech, China), matrix metalloproteinase (MMP) MMP2 (1:1,000; #66366-1-Ig; Proteintech, China), MMP9 (1:500; #27306-1-AP; Proteintech, China), and GAPDH (1:20,000; #60004-1-Ig; Proteintech, China) at 4°C overnight. Thereafter, the membrane was exposed to HRP-conjugated secondary antibody (1:5,000; #SA00001-1; Proteintech, China), followed by the development of protein bands via chemiluminescent (ECL) substrate. Grey values were determined with ImageJ software.

### Colony Formation Assay

500 LNCaP and PC-3 cells were seeded per well onto a six-well plate. Cells were cultured lasting 2 weeks, with medium changed every 3 days. In the following 2 weeks, colonies were fixed through ice-cold 100% methanol for 20 min as well as stained by 0.1% crystal violet lasting 20 min at room temperature. Colony formation was quantified in accordance with the percentage of area coverage per well.

### Transwell Assay

Invasive capacity of LNCaP and PC-3 cells was investigated with Matrigel-Coated Transwell Chambers (BD, United States ). Cells were planted onto the upper chamber. Following overnight incubation, cells in the lower chamber were fixed by 4% paraformaldehyde as well as stained by 0.1% crystal violet. Invasive cells were counted under a microscope (Olympus, Japan).

### Wound Healing Assay

LNCaP and PC-3 cells were inoculated into a 6-well plate. A 200 ul pipette tip was adopted for making cell scratches perpendicular to the well plate. The cell culture medium was aspirated as well as the well plate which was rinsed to wash away the cell debris. Thereafter, serum-free medium was added. Following 0 and 24 h, images were acquired and the absorbance value at 570 nm wavelength was measured.

### Statistics

R software (version 3.6.1) was adopted for data processing. Differences between groups were investigated with student’s t test or Wilcoxon test. 95% confidence intervals (CIs) and hazards ratios (HRs) were computed with uni- and multivariate Cox regression analyses. Meanwhile, Kruskal–Wallis and one-way ANOVA tests were applied for differential analyses among three groups. Correlation analyses between variables were conducted with Pearson or Spearman tests. Survival curves of overall survival (OS), disease-specific survival (DSS), and progression-free interval (PFI) were depicted utilizing log-rank and Kaplan-Meier tests. The area under the receiver operating characteristic curves (AUCs) of time-dependent ROC analyses were conducted for detection of the predictive potential of signature at different time points with timeROC package. *p* values < 0.05 were indicative of statistical significance.

## Results

### Deregulated Expression and Prognostic Significance of Ageing-Relevant Genes in Prostate Cancer

**FIGURE 1:**
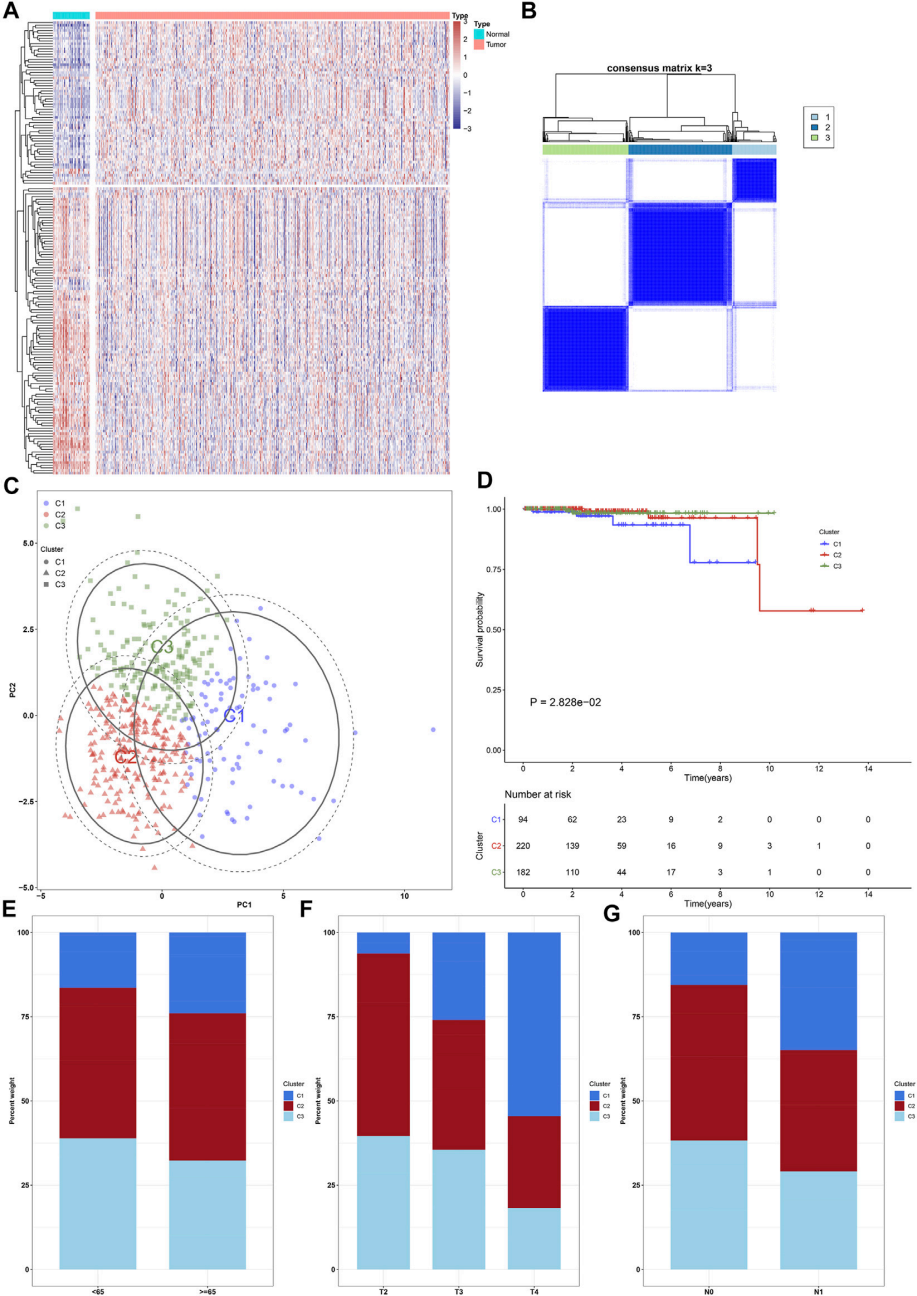
Characterization of ageing-relevant subtypes in prostate cancer based on the expression matrix of prognostic and specific ageing-relevant molecules. **(A)** Heatmap visualizing the expression of prostate cancer-specific ageing-relevant molecules in prostate tumors and controls. **(B)** Consensus matrix when k = 3 across prostate cancer patients in line with the expression matrix of prognostic prostate cancer-specific ageing-relevant molecules. **(C)** PCA plots verifying the dissimilarity among diverse ageing-relevant subtypes on the basis of the expression matrix of prognostic prostate cancer-specific ageing-relevant molecules. **(D)** Kaplan-Meier curves of OS among three ageing-relevant subtypes. **(E–G)** Bar plots of the distribution of diverse ageing-relevant subtypes across distinct clinical phenotypes, containing **(E)** age< 65 and ≥65 **(F)** pathological T2, T3, and T4 stage; **(G)** pathological N0 and N1 stage.

For assessment of the biological functions of ageing-relevant molecules in tumorigenesis and progression of prostate cancer, this study conducted a systematical investigation of the expression of ageing-relevant molecules between prostate tumors and controls in TCGA cohort. Consequently, there were 107 ageing-relevant genes with prominent down-regulations as well as 64 ageing-relevant genes with prominent up-regulations in prostate cancer compared with controls (Figure 1A; Supplementary Table S2). Additionally, we investigated the prognostic significance of these prostate cancer-specific ageing-relevant molecules. In total, 21 prognostic ageing-relevant genes presented prominent associations with prostate cancer prognosis (Table 1).

**TABLE 1:** Prognostic ageing-relevant genes in prostate cancer.

Ageing-relevant genes	Hazard ratio	95% lower hazard ratio	95% upper hazard ratio	*p*-value
NFKB2	3.5518	1.0444	12.0793	0.0424
BUB1B	2.2277	1.065	4.65962	0.0334
AIFM1	3.6733	1.1312	11.9284	0.0304
CCNA2	2.3025	1.2334	4.29813	0.0088
HSPD1	3.8693	1.4078	10.6345	0.0087
H2AFX	3.2532	1.212	8.73191	0.0192
SIRT7	15.42	2.1808	109.04	0.0061
HOXB7	2.316	1.2445	4.30993	0.008
MYC	1.9809	1.0054	3.90315	0.0482
TCF3	5.1575	1.2523	21.2403	0.0231
FEN1	4.4559	1.3481	14.7284	0.0143
DLL3	2.9151	1.1146	7.62379	0.0292
CDK1	1.8404	1.0403	3.2559	0.0361
MAX	17.976	1.1696	276.265	0.0382
HESX1	4.4069	1.4597	13.3043	0.0085
E2F1	2.194	1.0957	4.39324	0.0266
RAD51	2.2977	1.2174	4.3366	0.0103
TRAP1	3.7243	1.041	13.3246	0.0432
BLM	3.1899	1.1925	8.53262	0.0209
EGR1	0.615	0.4008	0.94355	0.026
BUB3	6.9116	1.6592	28.7915	0.0079

### Characterization of Ageing-Relevant Subtypes in Prostate Cancer With Diverse Clinical Prognosis

Herein, k = 3 was determined with the optimal clustering stability ranging from k = 2 to 9 in accordance with the similarity presented through the expression of 21 prognostic ageing-relevant genes and the proportions of ambiguous clustering method (Figure 1B). In total, 496 prostate cancer patients were clustered into three subtypes, named as C1 (*n* = 94), C2 (*n* = 220), and C3 (*n* = 182). Thereafter, PCA further confirmed three prominently diverse subtypes (Figure 1C). Survival analyses revealed that prognosis remarkedly differed among three ageing-relevant subtypes, and C1 presented the worst clinical prognosis (Figure 1D). Additionally, we investigated the discrepancy in ageing-relevant subtypes across distinct clinical phenotypes including age, T stage, as well as N stage (Figures 1E-G). Patients with advanced stages presented more proportions of C1.

### Landscape of Somatic Mutations and Copy-Number Alterations Across Prostate Cancer From Diverse Ageing-Relevant Subtypes

**FIGURE 2:**
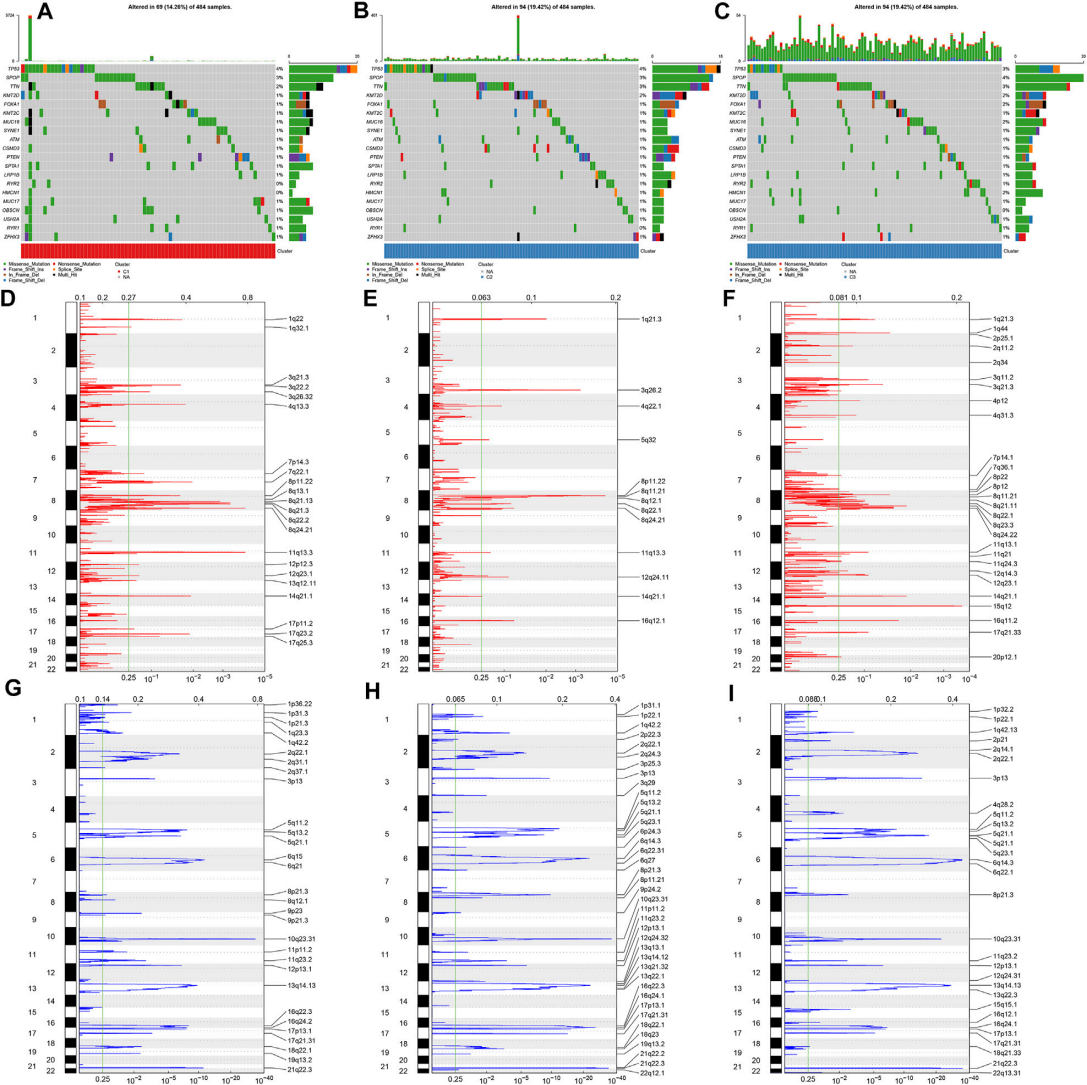
Overview of somatic mutations and copy-number alterations across prostate cancer from diverse ageing-relevant subtypes. **(A–C)** Oncoplot presented the somatic mutational landscape of prostate cancer patients in ageing C1, C2, and C3. Genes were ranked in accordance with their mutational frequencies. Side bar plots showed log10 converted q values calculated through MutSigCV. GISTIC2.0 identifying **(D–F)** amplification and **(G–I)** deletion across prostate cancer specimens in ageing C1, C2, and C3. The genome was oriented vertically from top to bottom and the GISTIC2.0 q-value at each locus was drawn from left to right with a log scale. The green line represented the significance threshold (q-value = 0.25).

The discrepancy in genetic mutations was investigated across diverse ageing-relevant molecular subtypes. As a result, a lower somatic frequency was noticed in ageing subtype C1 (69; 14.26%; Figure 2A) compared with C2 (94; 19.42%; Figure 2B) and C3 (94; 19.42%; Figure 2C). A few genes presented mutations shared by three subtypes such as TP53, SPOP, TTN, KMT2D, and FOXA1. Thereafter, SCNAs were compared among these subtypes for investigation of the genetic alterations. GISTIC2.0 analyses revealed that incidence of amplification presented higher frequencies in ageing subtype C3 in comparison to C1 and C2 (Figures 2D–F). Meanwhile, higher frequencies of deletion incidence were detected in ageing subtype C2 compared with C1 and C3 (Figures 2G–I).

### Characterization of Immune Landscape Across Diverse Ageing-Relevant Subtypes

**FIGURE 3:**
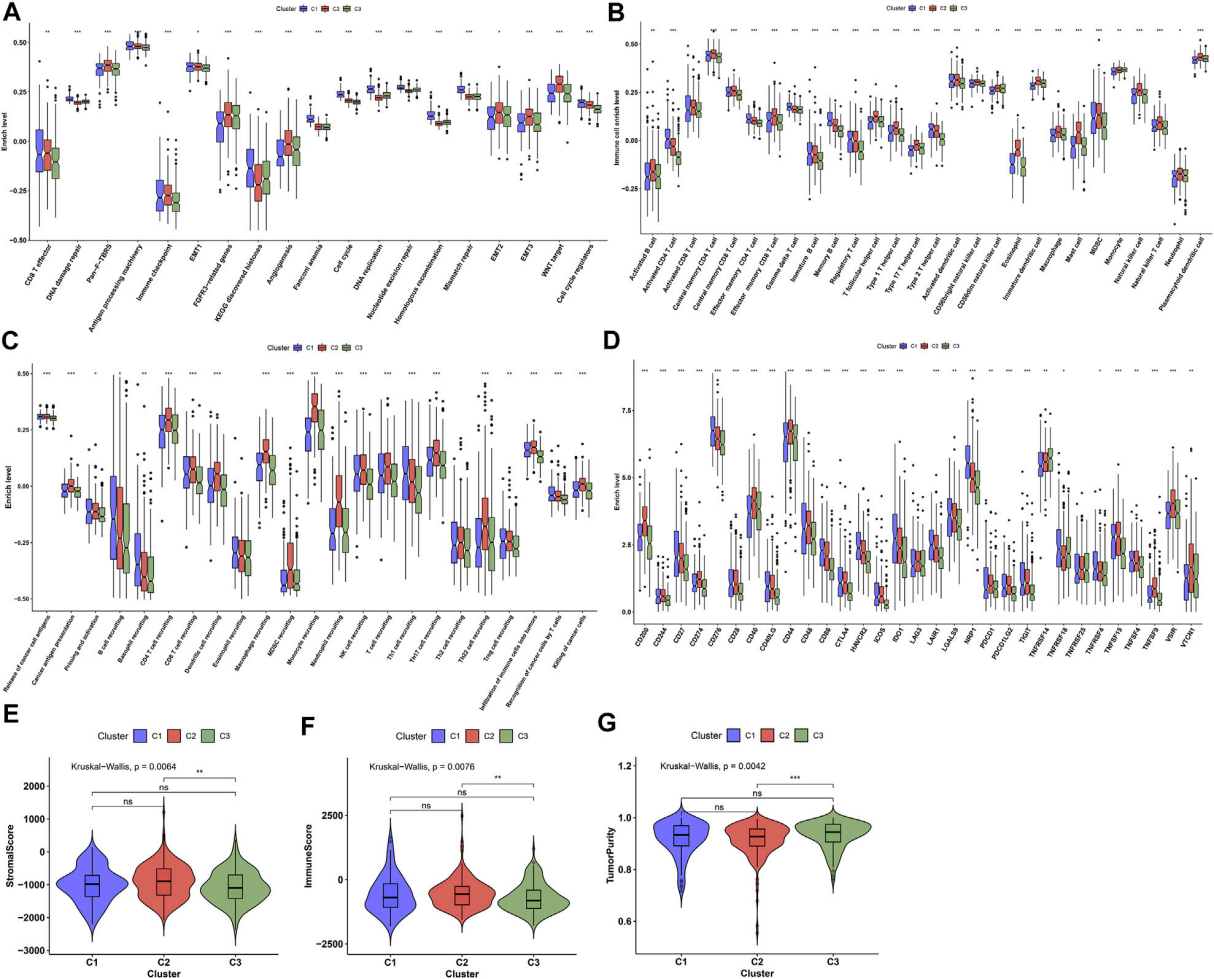
Characterization of immune landscape across diverse ageing-relevant subtypes. **(A)** Distribution of the activities of known biological processes across diverse ageing-relevant subtypes C1, C2, and C3. **(B)** Discrepancy in tumor microenvironment-infiltrating immune cells across diverse ageing-relevant subtypes. **(C)** Discrepancy in the activities of each step within cancer immunity cycle across distinct ageing-relevant subtypes. **(D)** Comparison of the expression of immune checkpoint molecules in distinct ageing-relevant subtypes. **(E–G)** Comparison of stromal and immune score and tumor purity in distinct ageing-relevant subtypes. Ns: no significance; *p values < 0.05; **p values < 0.01; and ***p values < 0.001.

The mechanisms underlying the discrepancy of three ageing-relevant subtypes were explored in depth. In Figure 3A, C1 presented increased activities of CD8^+^ T effector, DNA damage repair, antigen processing machinery, immune checkpoint, KEGG discovered histones, Fanconi anemia, cell cycle, DNA replication, nucleotide excision repair, homologous recombination, mismatch repair, and cell cycle regulators compared with C2 and C3, indicative of immune and tumorigenic activation in C1. Meanwhile, C2 showed the features of increased activities of pan-F-TBRS, EMT1-3, FGFR3-related genes, angiogenesis, and WNT target, indicative of stromal activation in C2. Our ssGSEA results demonstrated the prominent discrepancy in the infiltrations of immune subpopulations among three ageing-relevant subtypes. In detail, C1 presented the greatest infiltrations of activated CD4^+^ T cell, effector memory CD4 T cell, gamma delta T cell, immature B cell, and memory B cell while activated B cell, activated CD8^+^ T cell, central memory CD4 and CD8 T cell, effector memory CD8^+^ T cell, T follicular helper cell, type 1 helper cell, type 1 helper cell, activated dendritic cell, CD56bright natural killer cell, eosinophil, immature dendritic cell, macrophage, mast cell, MDSC, natural killer cell, natural killer T cell, and plasmacytoid dendric cell were remarkedly activated in C2 (Figure 3B). Nearly all immune subpopulations showed low infiltrations in C3. Thereafter, we investigated the activities of each step within the cancer immunity cycle. Compared with C1 and C2, C3 presented the lowest activities of each step within the cancer immunity cycle (Figure 3C), indicative of immune suppressive status in C3. Additionally, we noticed the increased expression of immune checkpoints in C1 and C2 in comparison to C3 at the transcriptional levels (Figure 3D). The ESTIMATE method was then adopted for detection of the overall infiltrations of stromal and immune cells within prostate cancer tissues. Consequently, C3 presented remarkedly reduced stromal and immune score as well as increased tumor purity in comparison to C2 (Figures 3E–G).

### Establishment of Co-expression Modules and Identification of Ageing-Derived Genes

**FIGURE 4:**
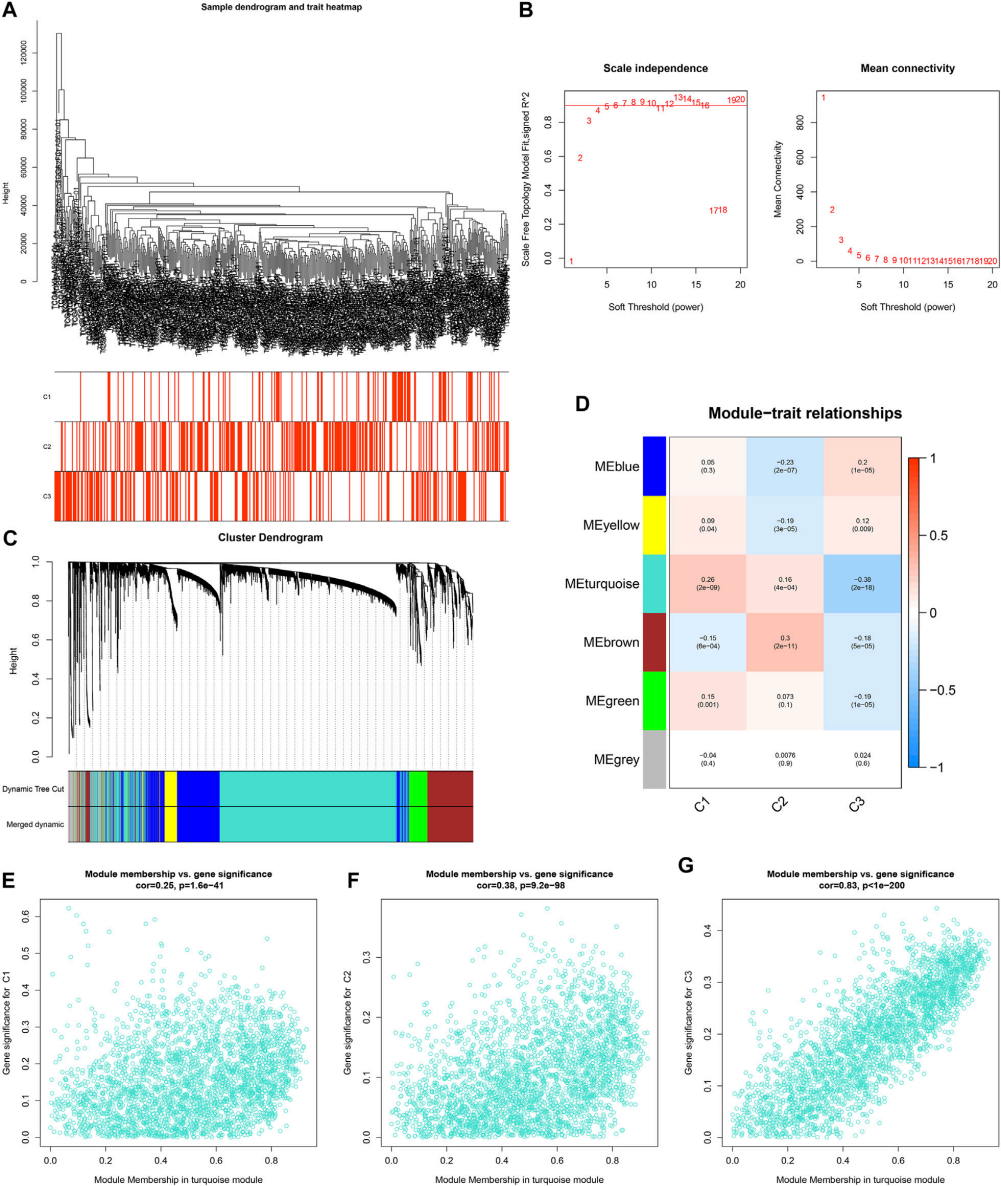
Establishment of co-expression modules and identification of ageing-derived genes. **(A)** Clustering dendrograms of prostate cancer specimens as well as heatmap of ageing-relevant subtypes. The clustering was conducted in accordance with the expression profiling of prostate cancer-specific ageing-relevant molecules. **(B)** Analyses of scale-free fit index (left) as well as mean connectivity (right) across diverse soft thresholds (power). **(C)** Dendrograms of prostate cancer-specific ageing-relevant molecules clustered in accordance with 1-TOM together with assigned module colors. **(D)** Heatmap depicting the associations of module eigengenes with ageing-relevant subtypes across prostate cancer. Each cell contained the Pearson correlation coefficient and *p* value. **(E–G)** Scatter plots depicting the interactions of module membership of turquoise with gene significance for ageing-relevant subtypes C1, C2, and C3.

For uncovering the key module most associated with ageing-relevant subtypes, we conducted WGCNA for identifying relevant intramodular hub genes utilizing the top-5000 variation genes in transcriptome profiling to establish a co-expression network (Figure 4A). The optimal soft thresholding value was determined when scale independence = 0.85 (Figure 4B). Thereafter, six co-expression modules were conducted (Figure 4C). In accordance with the heatmap of module-trait interactions, the turquoise module showed the strongest associations with ageing-relevant subtypes (Figure 4D). Further analyses confirmed that the genes in the turquoise module presented prominent interactions with each ageing-relevant subtype (Figures 4E–G), which were regarded as ageing-derived genes.

### Generation of an Ageing-Derived Gene Signature in Prostate Cancer Prognosis

**FIGURE 5:**
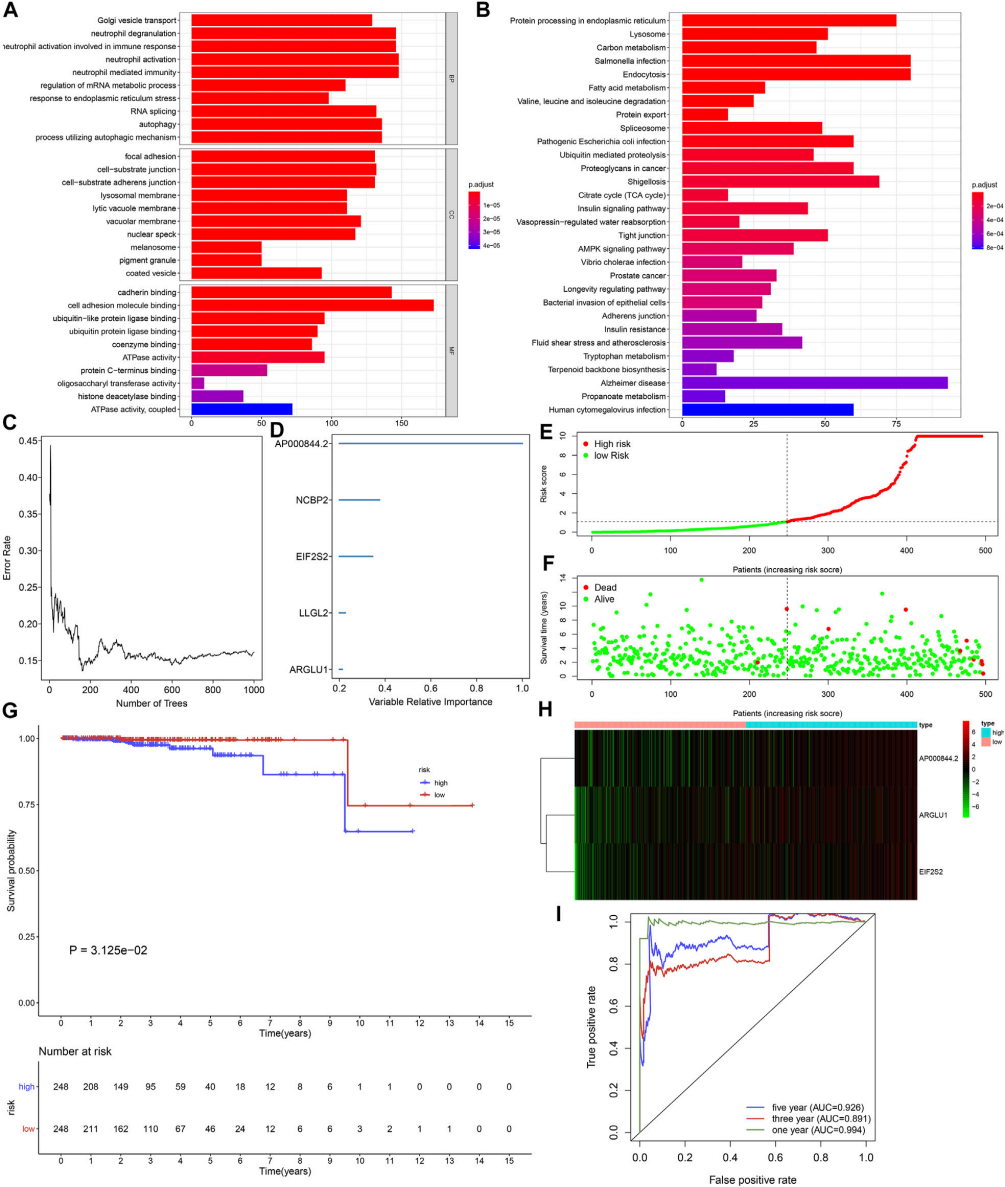
Generation of an ageing-derived gene signature in prostate cancer prognosis. **(A,B)** GO and KEGG enrichment results of ageing-derived genes. **(C)** Screening the most important ageing-derived genes with random forest algorithm. **(D)** The most important ageing-derived genes ordered by relative importance. **(E)** Distribution of ageing-derived risk scores across prostate cancer and determination of the optimal cutoff (dotted line). **(F)** Distribution of alive and dead status in high- and low-risk prostate cancer specimens. **(G)** Kaplan-Meier curves of OS in high- and low-risk prostate cancer patients. **(H)** Heatmap of the expression of ageing-derived molecules in high- and low-risk prostate cancer specimens. **(I)** The time-independent ROC analyses of ageing-derived gene signature for prediction of OS rate.

Functional enrichment and pathway analyses uncovered that ageing-derived genes exerted critical roles in modulation of immune response, autophagy, metabolism, and tumorigenic pathways (Figure 5). Univariate cox regression models were conducted and identified 43 prognostic ageing-derived genes in prostate cancer (Table 2). With the random forest algorithm, we determined the most important ageing-derived genes (Figure 5C), containing AP000844.2, NCBP2, EIF2S2, LLGL2, and ARGLU1 (Figure 5D). Following multivariate cox regression analyses, an ageing-derived gene signature was conducted in accordance with the following formula: risk score = ARGLU1 expression * 1.744375333 + EIF2S2 expression * 3.788531914 + AP000844.2 expression * 0.902670804. Thereafter, we calculated the risk score of each prostate cancer patient. With the optimal cutoff, patients were clustered into high- and low-risk groups (Figures 5E,F). High-risk patients showed remarkedly poorer OS outcomes in comparison to low-risk patients (Figure 5G). Heatmap depicted the discrepancy in expression of ARGLU1, EIF2S2, and AP000844.2 between two groups (Figure 5H). ROCs at one-, three-, and 5-year OS were 0.994, 0.891, and 0.926, confirming the excellent performance in prediction of prostate cancer prognosis (Figure 5I). We further externally validated the ageing-derived gene signature in the GSE116918 cohort. With the same formula, the risk score of each prostate cancer patient was calculated. With the optimal cutoff, we separated patients into high- and low-risk groups (Supplementary figure S1A). The survival status of two groups was shown in Supplementary figure S1B. As expected, the high-risk group had worse prognoses in comparison to the low-risk group (Supplementary figure S1C). The differences in expression of ARGLU1, EIF2S2, and AP000844.2 were found between two groups (Supplementary figure S1D). ROC results confirmed the prediction reliability of this signature (Supplementary figure S1E).

**TABLE 2:** Univariate cox regression models identify prognostic ageing-derived genes in prostate cancer.

Gene	Hazard ratio	95% lower hazard ratio	95% upper hazard ratio	*p*-value
UBE2G2	7.2936	1.8721	28.416	0.0042
LLGL2	4.8684	1.7541	13.5123	0.0024
RBM39	10.672	1.9495	58.4269	0.0063
JPT1	3.8762	1.6451	9.13299	0.0019
HPRT1	5.264	1.6305	16.9945	0.0055
AKR1C3	1.7206	1.2253	2.41606	0.0017
PBLD	2.3125	1.3073	4.09069	0.004
AHSA1	16.991	2.3381	123.471	0.0051
NUP62	12.338	3.4745	43.8142	0.0001
FUS	10.779	2.3031	50.4438	0.0025
SAFB2	8.0749	1.8929	34.4468	0.0048
FN3KRP	5.2678	1.6996	16.3268	0.004
GSK3A	0.2602	0.1183	0.57232	0.0008
U2AF2	11.019	1.9076	63.6475	0.0073
PSEN2	0.1914	0.0594	0.61691	0.0056
SRC	6.5828	2.0253	21.3963	0.0017
CIZ1	12.208	3.1135	47.8696	0.0003
C9orf78	16.147	2.5047	104.099	0.0034
ARGLU1	4.1012	1.436	11.7128	0.0084
ASNS	5.8566	1.9116	17.9427	0.002
MTA1	8.2451	1.9219	35.3727	0.0045
NOP58	12.545	2.2895	68.7369	0.0036
NELFCD	10.592	1.8148	61.8221	0.0087
SRSF2	22.697	3.4285	150.256	0.0012
ATF5	4.0599	1.8985	8.68196	0.0003
HSPD1	3.8707	1.4085	10.6374	0.0087
LUC7L3	10.554	2.6898	41.4131	0.0007
POLE3	10.511	3.1789	34.752	0.0001
GNL3	8.0334	1.9434	33.2082	0.004
PPIF	6.7803	1.6299	28.2051	0.0085
EIF2S2	13.488	1.9619	92.7209	0.0082
AL354710.2	2.5405	1.4834	4.35092	0.0007
YBX1	15.129	2.6738	85.6005	0.0021
PNN	5.9927	1.5837	22.6766	0.0084
AP000844.2	2.3478	1.509	3.65277	0.0002
SLBP	5.0714	1.7411	14.7722	0.0029
NCBP2	19.962	2.9001	137.399	0.0024
BNIP3	3.609	1.4275	9.12449	0.0067
H2AFZ	3.8894	1.4336	10.5519	0.0076
SHMT2	5.3828	1.8629	15.5537	0.0019
SAPCD2	3.6005	1.4214	9.12058	0.0069
RPS6KA4	6.0299	2.0624	17.6299	0.001
TDP2	5.3281	1.5989	17.7552	0.0064

### Ageing-Derived Gene Signature Acts as a Robust Prognostic Factor of Prostate Cancer

**FIGURE 6:**
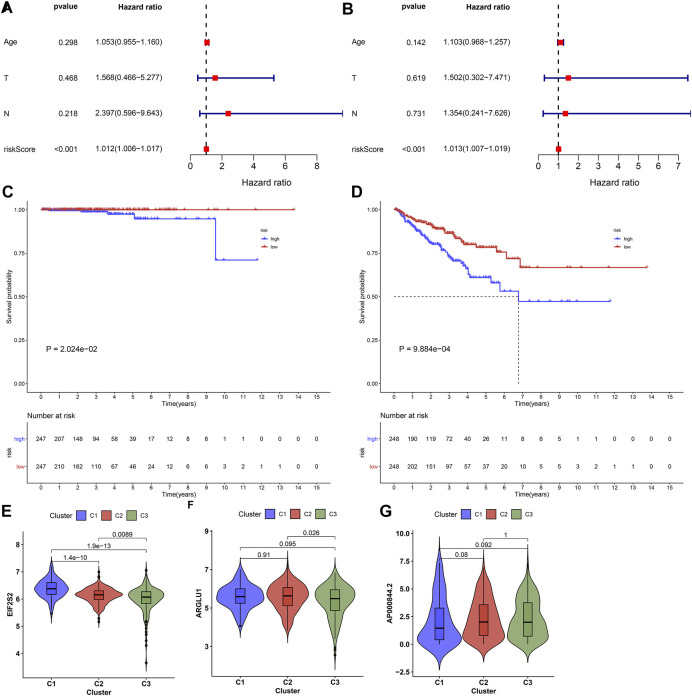
Ageing-derived gene signature acts as a robust prognostic factor of prostate cancer. **(A,B)** Uni- and multivariate cox regression models for the interactions of ageing-derived risk score, age, T stage, and N stage with prostate cancer prognosis. **(C,D)** Kaplan-Meier curves of DSS and PFI for high- and low-risk prostate cancer patients. **(E–G)** Distribution of the expression of EIF2S2, ARGLU1, and AP000844.2 across diverse ageing-relevant subtypes C1, C2 and C3.

Following uni- and multivariate cox regression analyses, ageing-relevant risk score may independently predict prostate cancer patients’ prognosis (Figures 6A,B). Additionally, we investigated that high-risk patients presented more dismal DSS and PFI outcomes in comparison to low-risk patients (Figures 6C,D). The expression of EIF2S2, ARGLU1, and AP000844.2 had the prominent discrepancy across diverse ageing-relevant subtypes (Figures 6E–G).

### Activated Pathways Involving Ageing-Derived Gene Signature

**FIGURE 7:**
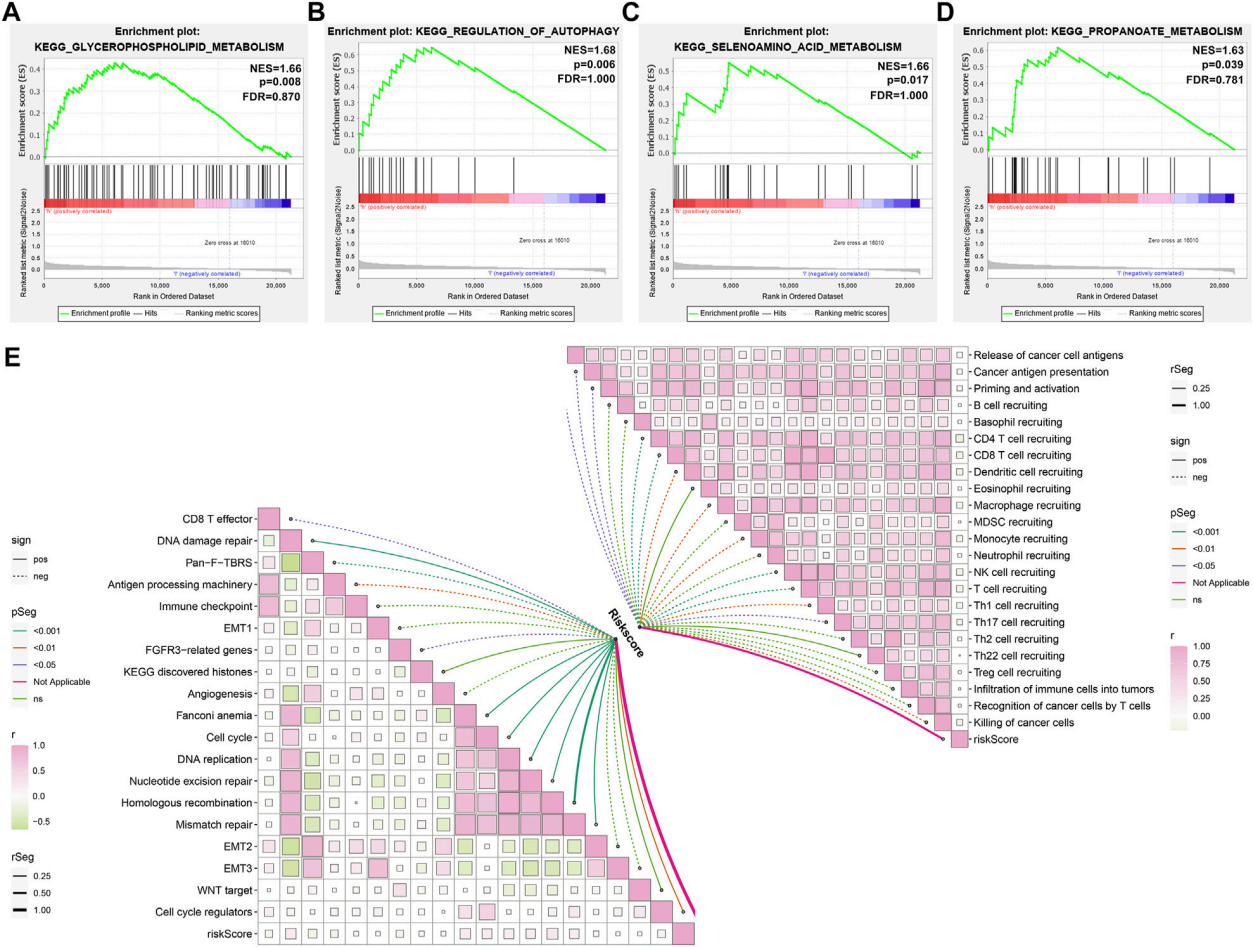
Activated pathways involving ageing-derived gene signature. **(A–D)** GSEA identifying the prominent KEGG pathways enriched by ageing-derived risk score, containing **(A)** glycerophospholipid metabolism **(B)** regulation of autophagy, **(C)** selenoamino acid metabolism, and **(D)** propanoate metabolism. **(E)** Interactions of ageing-derived risk score with activities of known biological processes and each step within the cancer immunity cycle.

GSEA uncovered that glycerophospholipid metabolism, regulation of autophagy, selenoamino acid metabolism, and propanoate metabolism presented higher activities in the high-compared with the low-risk group (Figures 7A–D). Additionally, ageing-derived risk score presented negative correlations to activities of CD8^+^ T effector, pan-F-TBRS, antigen processing machinery, and FGFR3-related genes but had positive correlations to activities of DNA damage repair, Fanconi anemia, cell cycle, DNA replication, nucleotide excision repair, homologous recombination, mismatch repair, and cell cycle regulators (Figure 7E). Moreover, we noticed that ageing-relevant risk score presented remarkedly negative associations with activities of most steps within the cancer immunity cycle (Figure 7E). The above data indicated that ageing-relevant risk score showed negative associations with immune and stromal activation.

### Associations of Ageing-Derived Gene Signature With Tumor Microenvironment-Infiltrating Immune Cells and Immune Response

**FIGURE 8:**
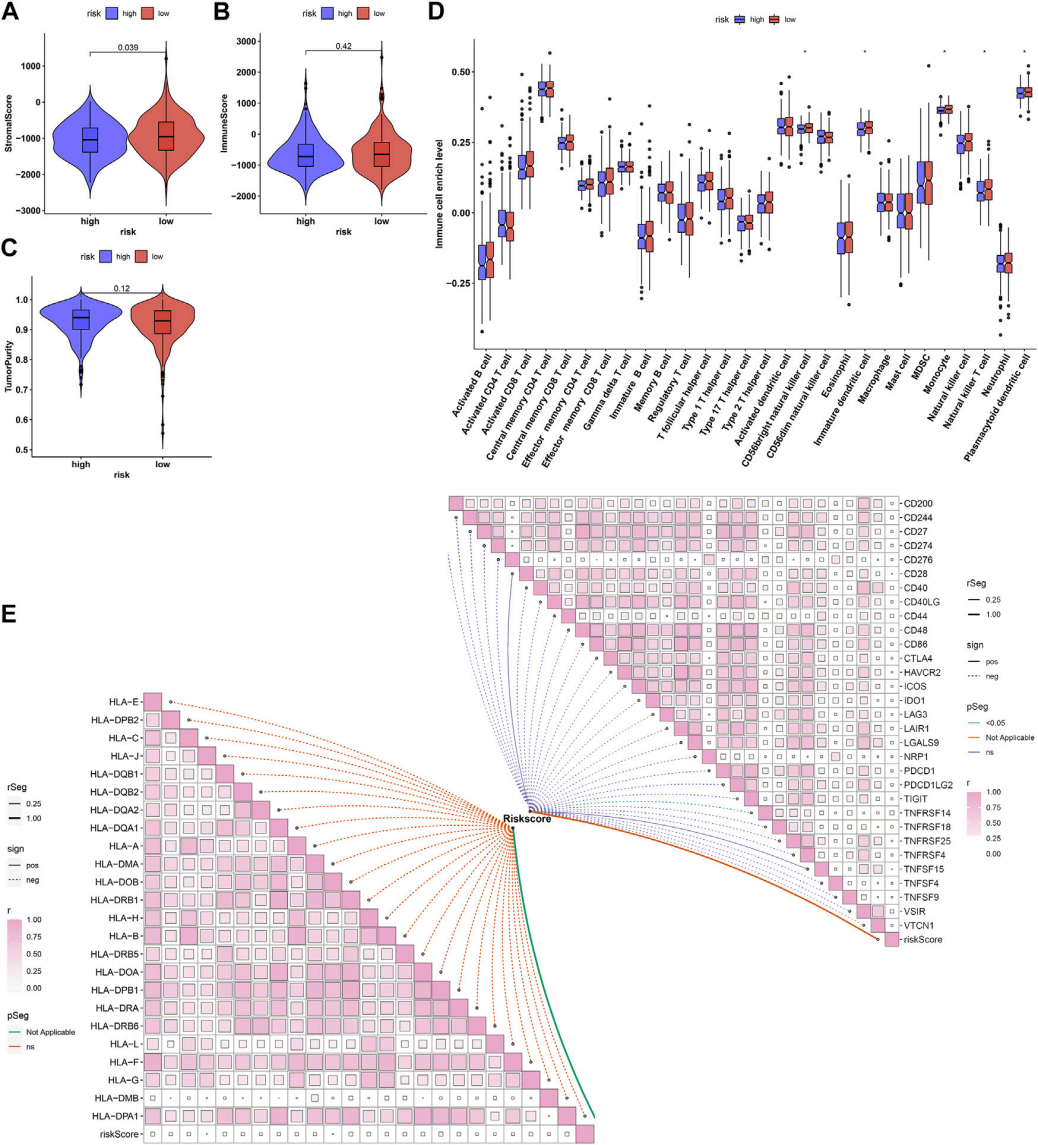
Associations of ageing-derived gene signature with tumor microenvironment-infiltrating immune cells and immune response. **(A–C)** ESTIMATE estimating the discrepancy in stromal and immune score as well as tumor purity in high- and low-risk groups. **(D)** The ssGSEA estimating the infiltrations of tumor microenvironment-infiltrating immune cells in high- and low-risk groups. *p values < 0.05. **(E)** Interactions of ageing-derived risk score with the expression of HLA and immune checkpoint molecules.

Further analyses showed that low-risk prostate cancer presented the prominent features of increased stromal and immune score as well as reduced tumor purity (Figures 8A–C). Analyses of tumor microenvironment-infiltrating immune cells revealed that CD56bright natural killer cell, immature dendritic cell, monocyte, natural killer T cell and plasmacytoid dendritic cell had remarkedly enhanced infiltration levels in low-risk patients (Figure 8D). Our in-depth correlation analyses uncovered the negative interactions of ageing-derived gene signature with HLA and immune checkpoint molecules (Figure 8E).

### EIF2S2 Up-Regulation Triggers Proliferation, Invasion and Migration of Prostate Cancer Cells

**FIGURE 9:**
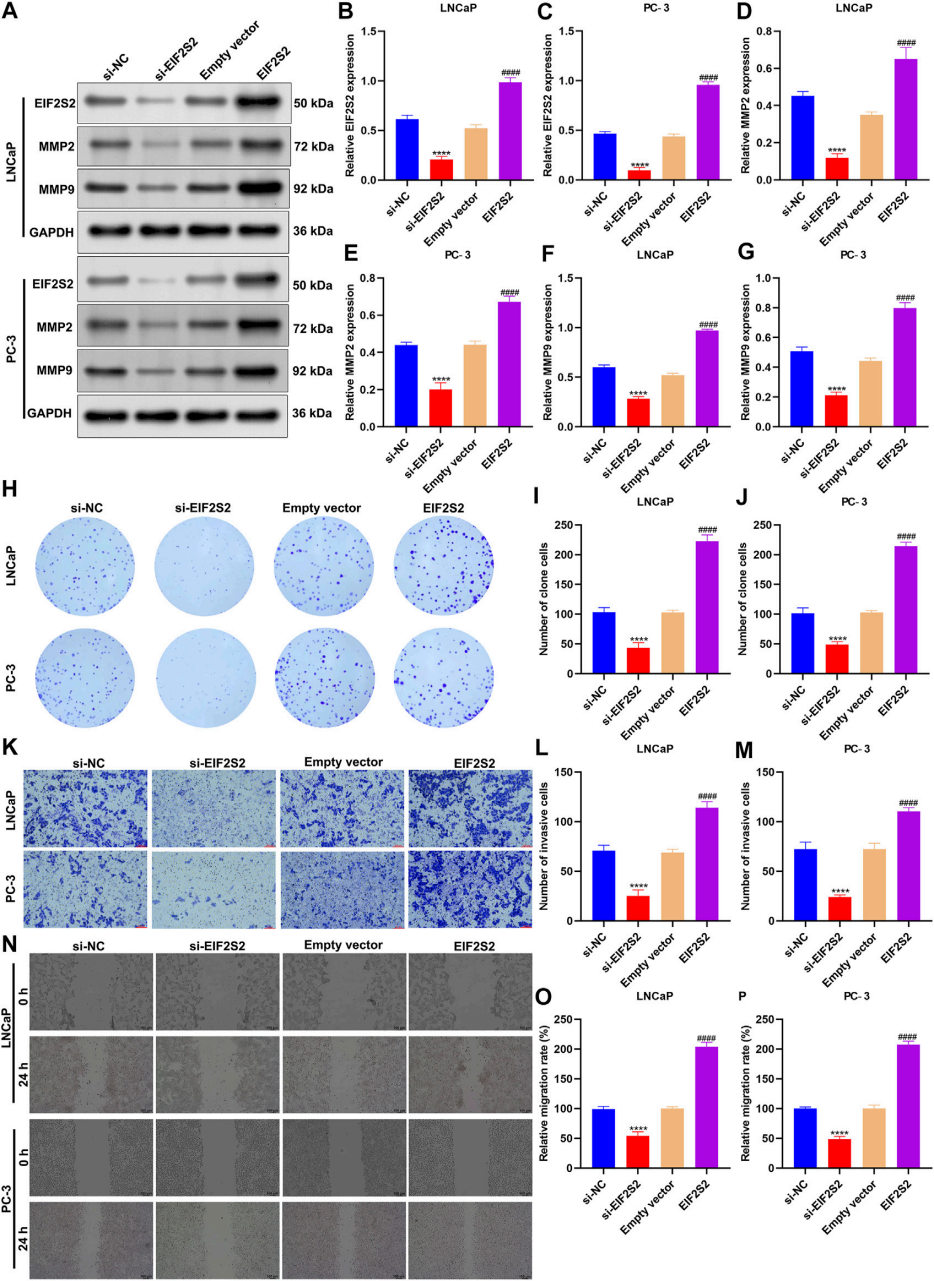
EIF2S2 up-regulation triggers proliferation, invasion, and migration of prostate cancer cells. **(A–G)** Western blotting for detections of the expression of EIF2S2, MMP2, and MMP9 in LNCaP and PC-3 cells after knocking-down or overexpressing EIF2S2. **(H–J)** Colony formation for evaluation of proliferative capacities of LNCaP and PC-3 cells after knocking-down or overexpressing EIF2S2. **(K–M)** Transwell for investigation of invasive abilities of LNCaP and PC-3 cells under knock-down or overexpression of EIF2S2. Scale bar = 5 μm; and magnification = ×200. **(N–P)** Wound healing for observation of migrated abilities of LNCaP and PC-3 cells under knock-down or overexpression of EIF2S2. Scale bar = 5 μm; and magnification = ×200. Compared with si-NC, ****p < 0.0001; compared with empty vector, ####*p* < 0.0001.

Among EIF2S2, ARGLU1, and AP000844.2, at present, there is still a lack of experimental evidence to confirm the role of EIF2S2 in prostate cancer. Therefore, we further verified the role of EIF2S2 in prostate carcinogenesis. EIF2S2 expression was separately remarkedly knock-downed or overexpressed in LNCaP and PC-3 cells (Figures 9A–C). Also, MMP2 and MMP9 expression was markedly decreased by knock-downed EIF2S2 while their expression was elevated when EIF2S2 was overexpressed (Figures 9D–G). Additionally, EIF2S2 knockdown reduced proliferation (Figures 9H–J), invasive (Figures 9K–M) as well as migrated (Figures 9N–P) abilities of LNCaP and PC-3 cells. The opposite findings were observed when EIF2S2 was overexpressed. These experimental evidences confirmed the carcinogenic roles of EIF2S2 in prostate cancer.

## Discussion

Because of the great heterogeneity among prostate cancer, OS rate and therapeutic response are both relatively low (Tolkach and Kristiansen, 2018; Wu et al., 2020). Hence, accurately identifying the molecular subtypes of prostate cancer is of importance to guide personalized treatment. Although increasing research has conducted a few molecular subtypes of prostate cancer, there is still considerable heterogeneity among the subtypes (Long et al., 2020; Meng et al., 2021; Song et al., 2021). Thus, more accurate classifications are urgently required for improving patients’ clinical prognosis. Through analysis of the alterations in ageing-relevant genes, critical clues may be gained for in-depth understanding the ageing process during prostate tumorigenesis at the transcriptional levels.

In this study, we characterized three ageing-relevant subtypes across prostate cancer based on the expression profiling ageing-relevant genes. There was a significant difference in expression patterns of ageing-relevant genes among three subtypes. Among them, subtype C1 presented the features of dismal clinical prognosis, low mutational frequency as well as immune activation; C2 was characterized by stromal and immune activation; and C3 showed immune suppressive status. Ageing may be triggered by accumulated cellular injury, led by genetic variations. Most prostate cancer is regarded as sporadic, primarily triggered by somatic variations (Xu et al., 2020). In-depth comprehending of somatic variations across prostate cancer contribute to new biomarkers regarding early screening, precision medicine, and clinical outcomes. The heterogeneity of tumor microenvironment containing cancer cells, stromal and infiltrating immune cells triggers diverse responses to immunotherapeutic therapy represented by immunological checkpoint inhibitors (Zeda Zhang et al., 2020). Tumor progression is a multistep process, which only involves the genetic and epigenetic variations within cancer cells. Nevertheless, many evidences demonstrated the critical roles of tumor microenvironment in tumor progression. Our data indicated that ageing process contributed to the tumor microenvironment of prostate cancer. The three ageing subtypes reflected the heterogeneity of the tumor microenvironment.

With WGCNA, we determined ageing-relevant subtype-associated co-expression model and genes. These ageing-derived genes exerted critical roles in modulation of immune response, autophagy, metabolism, and tumorigenic pathways. Among them, 43 ageing-derived genes had significant prognostic implications in prostate cancer. Evidences suggest that gene signatures are remarkedly and independently predictive of adverse pathology among men who present low-risk prostate cancer receiving prostatectomy (Cooperberg et al., 2021). Considering the influence of ageing on the heterogeneity of prostate cancer as well as clinical prognosis, an ageing-derived gene signature comprised of ARGLU1, EIF2S2, and AP000844.2 was conducted based on WGNCA, random forest, and uni- and multivariate cox regression models. Following verification, this signature was independently and robustly predictive of patients’ prognosis. The predictive value of this signature was also confirmed in an external dataset. Further analyses demonstrated that this signature showed negative interactions with immune suppression, which indirectly indicated the critical implication of ageing in immunotherapeutic effects. Previously, ARGLU1 acts as an important transcriptional coactivator as well as an important splicing regulator concerning stress hormone signals and developmental activation (Magomedova et al., 2019). ARGLU1 down-regulation is in relation to advanced TNM staging as well as more dismal OS of gastric cancer patients (Li et al., 2021). Additionally, overexpressed ARGLU1 reduces gastric cancer progression. EIF2S2 triggers tumorigenesis as well as progression through modulating MYC-mediated suppression by FHIT-relevant enhancer (Jiwei Zhang et al., 2020). The potential prognostic implications of AP000844.2 has been proposed in prostate cancer (Liu et al., 2020; Huang et al., 2021). Our experimental evidences demonstrated that EIF2S2 accelerated proliferation, invasion, and migration in prostate cancer cells, indicative of the tumorigenic function of EIF2S2 in prostate cancer.

Nevertheless, there are a few limitations in our study. Numerous prostate cancer specimens were required to verify the stability of ageing-relevant subtypes as well as the interactions of ageing with immunity require in-depth experimental verifications.

## Conclusion

Collectively, our research characterized three subtypes of ageing-relevant molecules for prostate cancer, indicative of diverse clinical outcomes. Ageing-relevant genes were critical contributors to the heterogeneity of the tumor microenvironment within prostate cancer. The ageing-relevant gene signature acted as a prospective predictor that presented great implications for distinguishing survival, ageing-relevant subtypes, tumor microenvironment cell infiltrating features, and immunotherapeutic responses of prostate cancer patients.

## Data Availability

The original contributions presented in the study are included in the article/Supplementary Material, further inquiries can be directed to the corresponding author.

## Abbreviations

TCGA, the Cancer Genome Atlas; PCA, principal component analyses; SCNA, somatic copy-number alteration; EMT, epithelial-mesenchymal transition; ssGSEA, single-sample gene-set enrichment analysis; ESTIMATE, Estimation of STromal and Immune cells in MAlignant Tumours using Expression; WGCNA, Weighted Gene Co-expression Network Analyses; GO, Gene Ontology; KEGG, Kyoto Encyclopedia of Genes and Genomes; GSEA, Gene Set Enrichment Analyses.

## References

[B1] Buckup M. Rice M. A. Hsu E.-C. Garcia-Marques F. Liu S. Aslan M. (2021). Plectin Is a Regulator of Prostate Cancer Growth and Metastasis. Oncogene 40 (3), 663–676. 10.1038/s41388-020-01557-9 33219316 PMC8078627

[B2] Calcinotto A. Kohli J. Zagato E. Pellegrini L. Demaria M. Alimonti A. (2019). Cellular Senescence: Aging, Cancer, and Injury. Physiol. Rev. 99 (2), 1047–1078. 10.1152/physrev.00020.2018 30648461

[B3] Charoentong P. Finotello F. Angelova M. Mayer C. Efremova M. Rieder D. (2017). Pan-cancer Immunogenomic Analyses Reveal Genotype-Immunophenotype Relationships and Predictors of Response to Checkpoint Blockade. Cel Rep. 18 (1), 248–262. 10.1016/j.celrep.2016.12.019 28052254

[B4] Cooperberg M. R. Cowan J. E. Lindquist K. J. Kobayashi Y. Simko J. P. Bengtsson H. (2021). Multiple Tissue Biomarkers Independently and Additively Predict Prostate Cancer Pathology Outcomes. Eur. Urol. 79 (1), 141–149. 10.1016/j.eururo.2020.09.003 33148472

[B5] Culp M. B. Soerjomataram I. Efstathiou J. A. Bray F. Jemal A. (2020). Recent Global Patterns in Prostate Cancer Incidence and Mortality Rates. Eur. Urol. 77 (1), 38–52. 10.1016/j.eururo.2019.08.005 31493960

[B6] de Magalhães J. P. Costa J. Toussaint O. (2004). HAGR: the Human Ageing Genomic Resources. Nucleic Acids Res. 33, D537–D543. 10.1093/nar/gki017 PMC539971 15608256

[B7] Ge R. Wang Z. Montironi R. Jiang Z. Cheng M. Santoni M. (2020). Epigenetic Modulations and Lineage Plasticity in Advanced Prostate Cancer. Ann. Oncol. 31 (4), 470–479. 10.1016/j.annonc.2020.02.002 32139297

[B8] Hänzelmann S. Castelo R. Guinney J. (2013). GSVA: Gene Set Variation Analysis for Microarray and RNA-Seq Data. BMC Bioinformatics 14, 7. 10.1186/1471-2105-14-7 23323831 PMC3618321

[B9] Haffner M. C. Zwart W. Roudier M. P. True L. D. Nelson W. G. Epstein J. I. (2021). Genomic and Phenotypic Heterogeneity in Prostate Cancer. Nat. Rev. Urol. 18 (2), 79–92. 10.1038/s41585-020-00400-w 33328650 PMC7969494

[B10] Huang H. Tang Y. Ye X. Chen W. Xie H. Chen S. (2021). The Influence of lncRNAs on the Prognosis of Prostate Cancer Based on TCGA Database. Transl Androl. Urol. 10 (3), 1302–1313. 10.21037/tau-21-154 33850764 PMC8039585

[B11] Jain S. Lyons C. A. Walker S. M. McQuaid S. Hynes S. O. Mitchell D. M. (2018). Validation of a Metastatic Assay Using Biopsies to Improve Risk Stratification in Patients with Prostate Cancer Treated with Radical Radiation Therapy. Ann. Oncol. 29 (1), 215–222. 10.1093/annonc/mdx637 29045551 PMC5834121

[B12] Jia K. Cui C. Gao Y. Zhou Y. Cui Q. (2018). An Analysis of Aging-Related Genes Derived from the Genotype-Tissue Expression Project (GTEx). Cell Death Discov. 4, 26. 10.1038/s41420-018-0093-y PMC6102484 30155276

[B14] Langfelder P. Horvath S. (2008). WGCNA: an R Package for Weighted Correlation Network Analysis. BMC Bioinformatics 9, 559. 10.1186/1471-2105-9-559 19114008 PMC2631488

[B15] Li F. Li J. Yu J. Pan T. Yu B. Sang Q. (2021). Identification of ARGLU1 as a Potential Therapeutic Target for Gastric Cancer Based on Genome-wide Functional Screening Data. EBioMedicine 69, 103436. 10.1016/j.ebiom.2021.103436 34157484 PMC8220577

[B16] Liberzon A. Birger C. Thorvaldsdóttir H. Ghandi M. Mesirov J. P. Tamayo P. (2015). The Molecular Signatures Database Hallmark Gene Set Collection. Cel Syst. 1 (6), 417–425. 10.1016/j.cels.2015.12.004 PMC4707969 26771021

[B17] Liu S. Wang W. Zhao Y. Liang K. Huang Y. (2020). Identification of Potential Key Genes for Pathogenesis and Prognosis in Prostate Cancer by Integrated Analysis of Gene Expression Profiles and the Cancer Genome Atlas. Front. Oncol. 10, 809. 10.3389/fonc.2020.00809 32547947 PMC7277826

[B18] Long X. Hou H. Wang X. Liu S. Diao T. Lai S. (2020). Immune Signature Driven by ADT-Induced Immune Microenvironment Remodeling in Prostate Cancer Is Correlated with Recurrence-free Survival and Immune Infiltration. Cell Death Dis 11 (9), 779. 10.1038/s41419-020-02973-1 32951005 PMC7502080

[B19] Magomedova L. Tiefenbach J. Zilberman E. Le Billan F. Voisin V. Saikali M. (2019). ARGLU1 Is a Transcriptional Coactivator and Splicing Regulator Important for Stress Hormone Signaling and Development. Nucleic Acids Res. 47 (6), 2856–2870. 10.1093/nar/gkz010 30698747 PMC6451108

[B20] Mariathasan S. Turley S. J. Nickles D. Castiglioni A. Yuen K. Wang Y. (2018). TGFβ Attenuates Tumour Response to PD-L1 Blockade by Contributing to Exclusion of T Cells. Nature 554 (7693), 544–548. 10.1038/nature25501 29443960 PMC6028240

[B21] Mayakonda A. Lin D.-C. Assenov Y. Plass C. Koeffler H. P. (2018). Maftools: Efficient and Comprehensive Analysis of Somatic Variants in Cancer. Genome Res. 28 (11), 1747–1756. 10.1101/gr.239244.118 30341162 PMC6211645

[B22] Meng J. Zhou Y. Lu X. Bian Z. Chen Y. Zhou J. (2021). Immune Response Drives Outcomes in Prostate Cancer: Implications for Immunotherapy. Mol. Oncol. 15 (5), 1358–1375. 10.1002/1878-0261.12887 33338321 PMC8096785

[B23] Mermel C. H. Schumacher S. E. Hill B. Meyerson M. L. Beroukhim R. Getz G. (2011). GISTIC2.0 Facilitates Sensitive and Confident Localization of the Targets of Focal Somatic Copy-Number Alteration in Human Cancers. Genome Biol. 12 (4), R41. 10.1186/gb-2011-12-4-r41 21527027 PMC3218867

[B24] Ritchie M. E. Phipson B. Wu D. Hu Y. Law C. W. Shi W. (2015). Limma powers Differential Expression Analyses for RNA-Sequencing and Microarray Studies. Nucleic Acids Res. 43 (7), e47. 10.1093/nar/gkv007 25605792 PMC4402510

[B25] Song J. Wang W. Yuan Y. Ban Y. Su J. Yuan D. (2021). Identification of Immune-Based Prostate Cancer Subtypes Using mRNA Expression. Biosci. Rep. 41 (1), BSR20201533. 10.1042/bsr20201533 33289508 PMC7785043

[B26] Subramanian A. Tamayo P. Mootha V. K. Mukherjee S. Ebert B. L. Gillette M. A. (2005). Gene Set Enrichment Analysis: a Knowledge-Based Approach for Interpreting Genome-wide Expression Profiles. Proc. Natl. Acad. Sci. 102 (43), 15545–15550. 10.1073/pnas.0506580102 16199517 PMC1239896

[B27] Swami U. McFarland T. R. Nussenzveig R. Agarwal N. (2020). Advanced Prostate Cancer: Treatment Advances and Future Directions. Trends Cancer 6 (8), 702–715. 10.1016/j.trecan.2020.04.010 32534790

[B28] Tolkach Y. Kristiansen G. (2018). The Heterogeneity of Prostate Cancer: A Practical Approach. Pathobiology 85 (1-2), 108–116. 10.1159/000477852 29393241

[B29] VanderWalde A. Hurria A. (2011). Aging and Osteoporosis in Breast and Prostate Cancer. CA: A Cancer J. Clinicians 61 (3), 139–156. 10.3322/caac.20103 21543824

[B30] Wang G. Zhao D. Spring D. J. DePinho R. A. (2018). Genetics and Biology of Prostate Cancer. Genes Dev. 32 (17-18), 1105–1140. 10.1101/gad.315739.118 30181359 PMC6120714

[B31] Wilkerson M. D. Hayes D. N. (2010). ConsensusClusterPlus: a Class Discovery Tool with Confidence Assessments and Item Tracking. Bioinformatics 26 (12), 1572–1573. 10.1093/bioinformatics/btq170 20427518 PMC2881355

[B32] Wu B. Lu X. Shen H. Yuan X. Wang X. Yin N. (2020). Intratumoral Heterogeneity and Genetic Characteristics of Prostate Cancer. Int. J. Cancer 146 (12), 3369–3378. 10.1002/ijc.32961 32159858

[B33] Xu H. Sun Y. You B. Huang C.-P. Ye D. Chang C. (2020). Androgen Receptor Reverses the Oncometabolite R-2-Hydroxyglutarate-Induced Prostate Cancer Cell Invasion via Suppressing the circRNA-51217/miRNA-646/TGFβ1/p-Smad2/3 Signaling. Cancer Lett. 472, 151–164. 10.1016/j.canlet.2019.12.014 31846689

[B34] Yang J. Jiang Q. Liu L. Peng H. Wang Y. Li S. (2020). Identification of Prognostic Aging-Related Genes Associated with Immunosuppression and Inflammation in Head and Neck Squamous Cell Carcinoma. Aging 12 (24), 25778–25804. 10.18632/aging.104199 33232279 PMC7803584

[B35] Yoshihara K. Shahmoradgoli M. Martínez E. Vegesna R. Kim H. Torres-Garcia W. (2013). Inferring Tumour Purity and Stromal and Immune Cell Admixture from Expression Data. Nat. Commun. 4, 2612. 10.1038/ncomms3612 24113773 PMC3826632

[B36] Yu G. Wang L.-G. Han Y. He Q.-Y. (2012). clusterProfiler: an R Package for Comparing Biological Themes Among Gene Clusters. OMICS: A J. Integr. Biol. 16 (5), 284–287. 10.1089/omi.2011.0118 PMC3339379 22455463

[B13] Zhang J. Li S. Zhang L. Xu J. Song M. Shao T. (2020). RBP EIF2S2 Promotes Tumorigenesis and Progression by Regulating MYC-Mediated Inhibition via FHIT-Related Enhancers. Mol. Ther. 28 (4), 1105–1118. 10.1016/j.ymthe.2020.02.004 32059763 PMC7132626

[B37] Zhang Z. Karthaus W. R. Lee Y. S. Gao V. R. Wu C. Russo J. W. (2020). Tumor Microenvironment-Derived NRG1 Promotes Antiandrogen Resistance in Prostate Cancer. Cancer Cell 38 (2), 279–296. 10.1016/j.ccell.2020.06.005 32679108 PMC7472556

