# Supplementary figures and images for: Comprehensive Characterization of Ageing-Relevant Subtypes Associated With Different Tumorigenesis and Tumor Microenvironment in Prostate Cancer
Source: Front Mol Biosci. 2022 Feb 21;9:803474. doi: 10.3389/fmolb.2022.803474 (PMC8898838; doi:10.3389/fmolb.2022.803474)

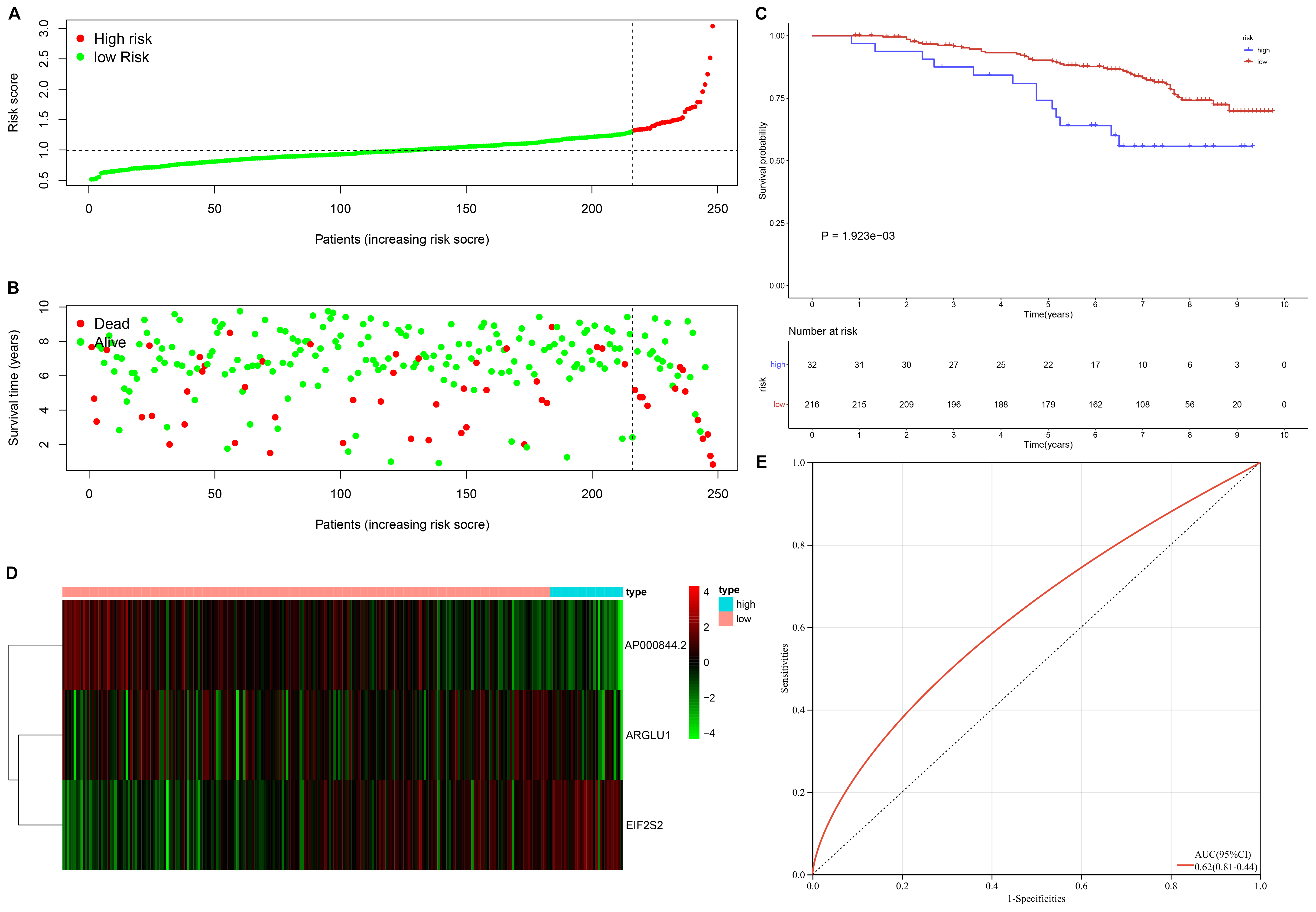

Supplement: Supplementary file 2 [file Image1.TIF]
